# Spontaneous Ventilation With High‐Flow Nasal Oxygen for Elective Suspension Microlaryngoscopy

**DOI:** 10.1002/oto2.54

**Published:** 2023-05-20

**Authors:** Cecile Courbon

**Affiliations:** ^1^ Department of Anesthesia University Hospital of Lausanne Lausanne Switzerland

**Keywords:** high‐flow nasal oxygen, spontaneous ventilation, tubeless anesthesia

## Abstract

**Objective:**

Spontaneous ventilation under intravenous anesthesia allows the surgeon to work without interruption or obstruction of the operating field during suspension microlaryngoscopy (SML). High‐flow nasal oxygen therapy (HFNO) is increasingly used in anesthesia. We hypothesized that its use during SML would increase patient safety even in situations where the airway is compromised by tumor or stenosis.

**Study Design:**

Retrospective observational study.

**Setting:**

University Hospital of Lausanne, Switzerland.

**Methods:**

Adults patients who were scheduled for elective microlaryngeal surgery and managed with HFNO in spontaneous ventilation under general anesthesia between October 2020 and December 2021.

**Results:**

Twenty‐seven patients for a total of 32 surgical procedures were performed under HFNO with spontaneous ventilation. Seventy‐five percent of the patients had respiratory symptoms. Twelve patients (42.9%) were planned for the treatment of subglottic or tracheal stenosis and 5 patients were managed for vocal cord cancer (18.5%). Out of 32 surgeries, 4 cases of saturation < 92% occurred, 3 of them during the decrease of the fraction of inspired oxygen to 30% for the use of the laser. In 3 cases, the patients were intubated to correct the hypoxemia.

**Conclusion:**

Spontaneous respiration using intravenous anesthesia and high‐flow nasal oxygen is a modern technique that increases patient safety while allowing the surgeon to work without interruption or imputation of the operative field during SML. This approach is particularly promising for the management of airways compromised by tumors or laryngotracheal stenosis.

Suspension microlaryngoscopy (SML) is an endoscopic technique performed under general anesthesia to assess the extent of endolaryngeal lesions, to perform biopsies, and to perform a possible treatment of these lesions. It is a diagnostic and/or therapeutic technique with important constraints: it is necessary to allow the surgeon to carry out the procedure in the best possible conditions and to give the necessary time while ensuring the safety of patients with significant comorbidities. The complexity of these procedures is based on the sharing of the airways between the anesthetist and the surgeon.^1^

Different anesthetic techniques are possible for SML.^2^, ^3^ Traditionally, general anesthesia with muscle relaxation and intubation is proposed. This technique allows for securing the airway and adequate ventilation. However, the presence of a tube potentially leads to obstruction of the operative site with a risk of airway fire in cases where cautery or laser is used. Alternatively, so‐called tube‐free techniques have been developed to overcome the problem of access to the surgical site.^2^ Among these techniques, spontaneous ventilation allows unlimited access to the endolaryngeal space but remains a challenge for the anesthetist who must ensure a sufficient level of anesthesia to tolerate the laryngeal suspension while maintaining spontaneous ventilation allowing adequate oxygenation.

In recent years, transnasal humidified rapid insufflation ventilatory exchange (THRIVE) delivered by high‐flow nasal cannula has been developed, first in intensive care units and more recently in the operating room.^4^ This technique of high‐flow nasal oxygenation (HFNO) has demonstrated its benefits in both apnea and spontaneous ventilation patients. In apnea, HFNO makes it possible to lengthen the time before the start of desaturation. In spontaneously ventilated patients, HFNO improves oxygenation, generates positive airway pressure, and reduces upper airway resistance. HFNO can be used in anesthesia during the induction phase as well as for apneic oxygenation during surgery. New fields of application are also emerging. However, its use in spontaneous ventilation in patients under general anesthesia is not often described.^5^

In our institution, we have opted for several years for tubeless techniques, such as jet ventilation, successive apnea with reventilation, or spontaneous ventilation. For this purpose, we use target‐controlled infusion (TCI) by propofol and remifentanil, which has the advantage of allowing a precise and rapid adaptation of the level of sedation to the degree of surgical stimulation.

Since the arrival of HFNO machines in our anesthesia department, we combine this oxygenation technique with our protocol of spontaneous ventilation under general anesthesia.

We describe here our experience of spontaneous ventilation under TCI for general anesthesia combined with HFNO for elective suspension microlaryngoscopy.

## Materials and Methods

This study was conducted at the University Hospital of Lausanne, Switzerland. The protocol was approved by the local ethics committee.

Between October 2020 and December 2021, suspension microlaryngoscopy was performed on 27 patients admitted for elective microlaryngoscopic surgery.

The inclusion criteria were: adult patients American Society of Anesthesiologists (ASA) 1 to 4 scheduled for microlaryngeal surgery (MLS) with an indication for spontaneous ventilation (difficult airway management and/or need for anesthesia without tube for the procedure). Previously tracheostomized patients, pregnant patients, or patients who refused to sign the general consent for research or were unable to do so, were excluded from the study. According to the protocols in force in our hospital, the patients did not receive premedication.

HFNO was delivered using an AIRcon respiratory humidifier base with an oxygen blender (WILAmed GmbH) which allows the inspired oxygen fraction to vary from 100% to 21% to prevent an airway fire in case of laser use.

During patient monitoring, preoxygenation via a high nasal flow cannula was performed with a flow rate of 30 L/min of 100% oxygen. After monitoring (electrocardiogram [ECG], SpO_2_, respiratory rate, noninvasive blood pressure), intravenous anesthesia (TCI) was started. The initial Ce concentration of propofol was 2 mcg/mL (Schnider model) and that of remifentanil was 1 ng/mL (Minto model). The concentrations were progressively increased by a step of 0.5 until the patient lost consciousness. Once unconsciousness was achieved, the flow of 100% oxygen was increased from 60 to 70 L/min. The concentrations of propofol and remifentanil were increased until there was no reaction to a vigorous jaw‐thrust maneuver. Direct laryngoscopy with a macintosh blade was performed and the epiglottis, vocal cords, and trachea were sprayed with a solution of oxybuprocaine 10 mg/mL. Local anesthesia was repeated every 30 minutes. The time of direct laryngoscopy corresponded to the transition from the induction to the maintenance phase. The concentration of remifentanil was slightly increased for the laryngeal suspension. The procedure was performed with spontaneous ventilation. In all cases, jet ventilation equipment was ready and variable sizes of tubes were available to replace HFNO if desaturation occurred. If the laser was used, the fraction of inspired oxygen (FiO_2_) was decreased to 30%. In the case of tracheal balloon dilatation, the concentration of remifentanil was transiently increased to achieve a short apnea in order to prevent inspiratory strain with a closed glottis until the end of the dilatation. The respiratory rate was monitored by ECG impedance. At the end of the surgery, the O_2_ flow was decreased to 20 L/min and then stopped at the time of the exit of the operating room. The time between the end of the surgery to the discharge from the operating room corresponds to the patient's recovery phase The anesthesia data (hemodynamic and respiratory data, drug concentrations) were entered manually on a monitoring sheet which was then stored in the hospital's computer system. The data useful for the study were then extracted manually from the computer system.

Data were analyzed using Stata 17.0 (StataCorp, 2021, Stata Statistical Software: Release 17: StataCorp LLC).

## Results

Between October 2020 and December 2021, 27 patients with 32 surgical procedures were performed under HFNO with spontaneous ventilation.

There were 16 females and 11 males with a mean (standard deviation, SD) age of 52.9 (14.2) years. The mean body mass index (BMI) (SD) was 27.1 (5.8). Half of the patients (51.9%) had an ASA 3 score. The most common associated comorbidities were coronary artery disease, sleep apnea syndrome, and hypertension (Table 1).

**Table 1:** Study Population Demographics (n = 27)

Sex, n (%)	
Female	11 (40.7)
Male	16 (59.3)
Age	
Mean (SD)	52.9 (14.2)
Median, LQ‐UQ, min‐max	56.0, 43.0‐64.0, 19.0‐76.0
ASA, n (%)	
1	1 (3.7)
2	12 (44.4)
3	14 (51.9)
BMI	
Mean (SD)	27.1 (5.8)
Median, LQ‐UQ, min‐max	26.8, 23.0‐30.4, 16.7‐42.5

Abbreviations: ASA, American Society of Anesthesiologists; BMI, body mass index; LQ, lower quartile; max, maximum; min, minimum; UQ, upper quartile.

**Figure 1:**
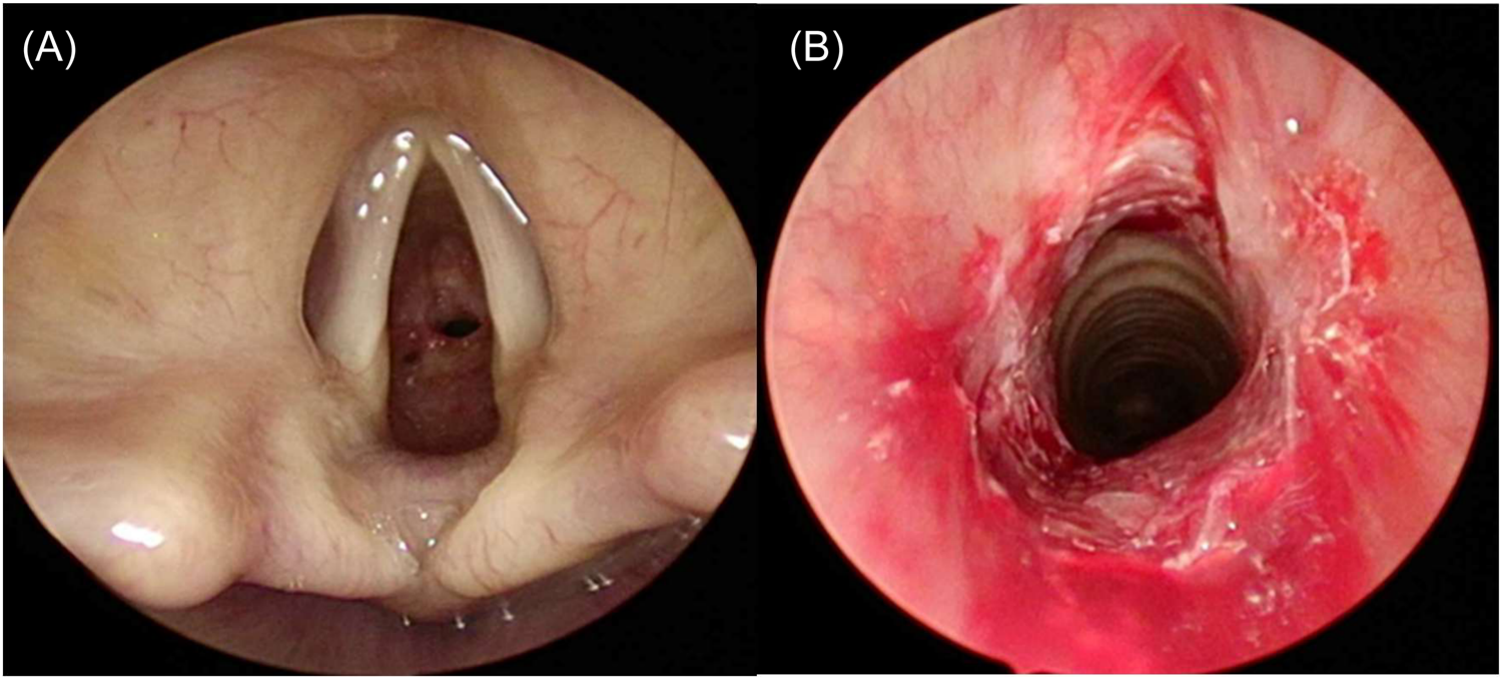
Example of a severe subglottis stenosis. (A) Before treatment. (B) After dilatation and kenacort® injection

Regarding surgical indications, 12 patients (42.9%) were planned for the treatment of subglottic or tracheal stenosis and 5 patients were managed for vocal cord cancer (18.5%). Seventy‐five percent of the patients had respiratory symptoms, most often dyspnea on exertion or at rest (13 patients, 48.1%). In 25% of cases, stridor was found on clinical examination. Finally, 4 patients had an oxygen saturation at rest of less than 95%. Only 1 patient required oxygen preoperatively due to subglottic stenosis of more than 90% of the tracheal lumen (Figure 1).

Thirty‐two operations were performed, with 1 patient having been treated 3 times for tracheal stenosis and 3 patients having been treated twice (redo of surgical margins). The surgery included 11 balloon tracheal dilatations, 9 laser procedures, 1 combined laser/dilatation procedure, 3 vocal cord medializations, 3 endoscopies for biopsies, and 5 control procedures (Table 2).

**Table 2:** Diagnosis and Surgery

Primary diagnosis, n (%)	
Subglottic or tracheal stenosis	12 (44.4)
Vocal cord cancer	5 (18.5)
Vocal cord paralysis	4 (14.8)
Laryngeal or tracheal lesion	5 (18.5)
Laryngeal spasm	1 (4)
Surgery, n (%)	
Dilatation	11 (34.3)
Laser	9 (28.1)
Laser/dilatation	1 (3.1)
Control procedure	5 (15.6)
Medialization	3 (9.4)
Biopsy	3 (9.4)

The median procedure time was 57 minutes (46.0‐69.5 [30.0‐153.0]) and the median surgical time was 40 minutes (30.5‐55.0 [8.0‐130.0]) (Table 3).

**Table 3:** Surgery Times

Duration of induction, min	
Mean (SD)	6.7 (4.1)
Median, LQ‐UQ, min‐max	5.0, 4.5‐8.0, 2.0‐21.0
Duration of surgery, min	
Mean (SD)	45.8 (25.6)
Median, LQ‐UQ, min‐max	40.0, 30.5‐55.0, 8.0‐130.0
Duration of awakening, min	
Mean (SD)	7.7 (4.1)
Median, LQ‐UQ, min‐max	8.0, 5.0‐9.0, 3.0‐26.0
Duration of intervention, min	
Mean (SD)	63.6 (28.6)
Median, LQ‐UQ, min‐max	57.0, 46.0‐69.5, 30.0‐153.0

Abbreviations: LQ, lower quartile; max, maximum; min, minimum; UQ, upper quartile.

Regarding anesthesia, the median propofol site concentration was 5.4 (4.0‐6.1 [3.3‐8.0]) and the median remifentanil concentration was 3.0 (2.5‐4.0 [1.3‐9.0]).

Out of 32 surgeries, 4 cases of saturation <92% occurred, 3 of them during the decrease of the FiO_2_ to 30% for the use of the laser. In 3 cases, the patients were intubated to correct the hypoxemia and then the tube was removed to continue surgery. Two episodes of desaturation requiring intubation occurred during 2 procedures in the same patient with class 1 obesity (BMI = 34.5) combined with a sleep apnea syndrome under continuous positive airway pressure. The other intubation occurred in a 70 years old man with chronic obstructive pulmonary disease and a preoperative saturation of 94%.

## Discussion

This study aimed to investigate the application, feasibility, and efficiency of Spontaneous respiration with intravenous anesthesia and high‐flow nasal oxygen for microlaryngeal surgery. Through these 32 cases of MLS, we have shown that high‐flow oxygenation with a nasal cannula was effective in maintaining airway permeability and oxygenation in spontaneous ventilation including during laser use, and allowed the surgeons to work without interruption.

Suspension microlaryngoscopy is a procedure in which the surgeon and anesthetist share the patient's airway. During this procedure, the traditional anesthesia with muscle relaxation and placement of an endotracheal tube can be an obstacle for the surgeon because the tube obstructs the operating field, especially if it concerns the posterior glottis or subglottis. Furthermore, in case of obstruction or severe stenosis, intubation may be difficult or impossible. To counteract these difficulties, anesthesia teams have developed tubeless anesthesia techniques,^2^, ^3^, ^6^ which have the advantage of offering unobstructed access to the larynx, subglottis, and trachea. One of the most common ways of doing this is to work under apnea with intermittent endotracheal tube ventilation. The surgeon operates during the apnea phases and then when the oxygen saturation reaches a predefined level the patient is reventilated. This technique requires the surgeon to interrupt the surgery regularly. In addition to this, repeated intubations can lead to lesions or even laryngeal edema.

Another technique of tubeless anesthesia is the use of automated high‐frequency jet ventilation. Automatic jet ventilators such as the Mistral or Monsoon (Acutronic Medical Systems) deliver a high‐pressure airflow into the airway through a cannula which can be placed transglottically or transtracheally or via a suppraglottic cannula. This technique usually requires muscle relaxation of the patient. Apart from the fact that these devices are not available in all centers, one of the main limitations to the use of jet ventilation is the presence of an obstruction to the air outflow as in the case of stenosis or obstructive mass, which leads to the possible occurrence of barotrauma.

Spontaneous ventilation under intravenous anesthesia allows permanent and complete access to the larynx, subglottis, and trachea, and allows uninterrupted surgery.^7^, ^8^ It is interesting for dynamic examination of the airways in order to study cord mobility or to look for a collapse. However, it requires perfect adequacy of the depth of anesthesia to the intensity of the surgical stimulus, and careful monitoring of the patient in order to avoid apneas and obstructions that could lead to desaturation. The possibility to ventilate the patient must be available at all times.

The HFNO is a device that provides a heated and humidified oxygen flow of more than 15 L/min via a nasal cannula. It also offers the possibility to vary the oxygen concentration.

Widely used in intensive care during hypoxemic respiratory distress and in postoperative care to reduce reintubation, HFNO is finding more and more indications in anesthesia. Used during induction in obese patients, HFNO during preoxygenation is more effective than the facemask in increasing the arterial partial pressure in oxygen. Similarly, during rapid sequence induction, HFNO can extend the apnea time without desaturation.^9^, ^10^

The effectiveness of THRIVE in prolonging secure apnea time during upper airway surgery has already been demonstrated in several studies with different anesthesia protocols including or not the use of paralytics.^11^, ^12^, ^13^, ^14^, ^15^, ^16^, ^17^

But few studies have investigated the advantage of combining spontaneous ventilation with the benefits of HFNO. Lee et al^18^ reported the use of this new airway management technique in the treatment of acute adult epiglottitis. Booth et al^5^ demonstrated that the association of spontaneous breathing and HFNO provided safety and good oxygenation in a retrospective study of elective nonlaser surgery. Our study shows the same advantages including laser resection.

The obstruction of the upper airways during induction of anesthesia is a well‐known phenomenon.^19^, ^20^ Propofol used in TCI induces progressive collapse^21^ and is now widely available at sleep endoscopy to demonstrate the different sites of pharyngeal collapse that characterize obstructive sleep apnea.^22^, ^23^ Except for testing the depth of anesthesia, no maneuvers on the upper airway have been necessary to maintain its permeability. We believe that the positive nasopharyngeal pressure generated by HFNO counteracts this tendency to obstruction.^24^

Habrial et al^25^ found that the presence of a laryngeal tumor was a cause of failure of panendoscopies in spontaneous ventilation and concluded that the use of HFNO could be interesting. In our series of suspension microlaryngoscopies, the presence of a laryngeal tumor was not an obstacle to the technique, possibly due to the use of HFNO.

Twelve patients were treated endoscopically for laryngotracheal stenosis. If spontaneous inspiration is known to generate negative pressure and aggravate the reduction of the tracheal lumen and inflow,^26^ Booth et al^5^ demonstrated that this phenomenon was counteracted when using HFNO. We found the same effect and in our center, the use of HFNO in spontaneous ventilation is indicated as the first line in the management of laryngotracheal stenosis.

This study has several limitations due to its retrospective and monocentric nature, the small number of included patients, and the lack of data concerning end‐tidal carbon dioxide. Although transcutaneous CO_2_ measurement is available in our operating rooms, it was not always used due to the time required for calibration and interferences.

## Conclusion

The use of spontaneous ventilation with HFNO under general anesthesia is a modern technique that increases patient safety while allowing the surgeon to work without interruption or imputation of the operative field during SML. It is an interesting alternative to anesthesia with intubation, successive apnea, or jet ventilation. This approach is particularly promising for the management of airways compromised by tumors or laryngotracheal stenosis, the incidence of which is expected to increase as a result of prolonged Covid‐related intubation.

## Author Contributions

The author confirms sole responsibility for the following: study conception and design, data collection, analysis and interpretation of results, and manuscript preparation.

## Disclosures

### Competing interests

The author has no conflicts of interest to declare.

### Funding source

The author has no funding.
